# Supplementary material for: Dietary diversity and association with non-communicable diseases (NCDs) among adult men (15–54 years): A cross-sectional study using National Family and Health Survey, India
Source: PLOS Glob Public Health. 2023 Apr 26;3(4):e0001775. doi: 10.1371/journal.pgph.0001775 (PMC10132668; doi:10.1371/journal.pgph.0001775)
Supplement: Attachment — Submitted filename: 3_Responses to Reviewers_r2.pdf [file pgph.0001775.s001.pdf]

Author’s Responses to the Editor(s) and Reviewers

First of all, I would like to express my sincere thanks to the editor and anonymous reviewer for reviewing the manuscript and providing very thoughtful suggestions. All the suggestions are well taken and incorporated in the revised text

Submission Cheek list:

1. A rebuttal letter that responds to each point raised by the editor and reviewer(s). You should upload this letter as a separate file labeled 'Response to Reviewers'.

   Author Response: Completed.

2. A marked-up copy of your manuscript that highlights changes made to the original version. You should upload this as a separate file labeled 'Revised Manuscript with Track Changes'.

   Author Response: Completed

3. An unmarked version of your revised paper without tracked changes. You should upload this as a separate file labeled 'Manuscript'.

   Author Response: Completed

Journal requirements:

1. Please review your reference list to ensure that it is complete and correct. If you have cited papers that have been retracted, please include the rationale for doing so in the manuscript text, or remove these references and replace them with relevant current references. Any changes to the reference list should be mentioned in the rebuttal letter that accompanies your revised manuscript. If you need to cite a retracted article, indicate the article’s retracted status in the References list and also include a citation and full reference for the retraction notice.

   Author Response: The reference list is now corrected.

2. Please provide separate figure files in .tif or .eps format only and remove any figures embedded in your manuscript file. Please also ensure that all files are under our size limit of 10MB.

   Author Response: Two separate figures are prepared in .tif format within the 10 MB size.

   Figure 1: *Food consumption patterns among adult men in India* ;

Figure 2 *Geospatial pattern of (a) mean dietary diversity, the prevalence of (b) diabetes, (c) heart diseases, and (d) cancer among men in India, 2015-16*

3. We do not publish any copyright or trademark symbols that usually accompany proprietary names, eg ©, ®, ™ (e.g. next to drug or reagent names). Please remove all instances of trademark/copyright symbols throughout the text, including ® on pages 24 and 25.
   Author Response: Corrections were made in the original text. All trademarks including ® have been removed from the manuscript.

4. Please provide a complete Data Availability Statement in the submission form, ensuring you include all necessary access information or a reason for why you are unable to make your data freely accessible. If your research concerns only data provided within your submission, please write "All data are in the manuscript and/or supporting information files" as your Data Availability Statement.

   Author Response: A data availability section is added to the manuscript (Section -7).

   "The dataset analyzed during the current study are available in the Demographic and Health Survey (DHS) repository at https://dhsprogram.com/data/available-datasets.cfm, and can be accessed on formal request."

Reviewer- 1 Comment and Author Responses:

1. Introduction needs to be shortened. Certain parts can be moved to discussion

   Author Response: We appreciate the comment. A paragraph is omitted from the 'introduction' section, and relevant parts are added to the 'discussions.'

2. Discussion, despite moving parts from introduction, still need to be concise

   Author Response: The comment is well accepted, and the 'discussion' part was made concisely as recommended.

3. Any reference to Dietary Diversity Score? Was based on prior work? Please clarify. Also a more clear description in introduction may be valuable as part of aims of the study

   Author Response: The Dietary Diversity Score was used in many studies to assess the diet diversity pattern. The appropriate references were added to the manuscript (Ref no 69, 70). Also, a clear description on the recall period that used to construct the DDS is

added. The detail methodology of dietary diversity score is mentioned in the methodology section (section 2.3) for more clarification.

4. Please clarify why specific interest in males, why not females, and what data that may require an independent study?

   Author Response: Though non-communicable diseases are the leading causes of death for both men and women worldwide. But the latest report in India (NFHS, 2019-20) shows that the prevalence of NCDs such as diabetes, hypertension, etc., are higher among men than women. Therefore, the present study has taken a specific interest in researching men. However, it could be an interesting comparative study if women's counterparts also are included.

5. Considering the multivariate analyses, suggest looking into a Bayesian model analysis.
   Author Response: In the present model, outcome variables are NCDs which are coded in binary i.e., yes or no. The model objective was to estimate the odds ratio of the occurrence of diabetes, heart diseases and cancer. Therefore, we believe that using binary logistic regression model will be the appropriate model fit for this study.

Reviewer- 2 Comments:

1. Reference for 'three-quarters of global NCD deaths occur in low- and middle-income countries.'

   Author Response: The author would like to thank the reviewer for highlighting the missing reference. The reference is added to the manuscript (see ref 71).

2. 'Noncommunicating diseases'- non-communicable

   Author Response: It is corrected in the text and used ‘non-communicable’ instead of ‘Noncommunicating diseases’

3. It would be more appropriate to replace 'heart disease' with coronary heart disease

   Author Response: The comment is well accepted. The term ‘heart disease is replaced with the term ‘coronary heart disease’ in relevant places

4. 'optimal growth' should be omitted

   Author Response: We would like to thank the editor for this correction. We have omitted the term ‘optimal growth’.

5. Explaining the process and reasoning for developing the DDS will help validate the system. As there is a confusion if the authors are trying to validate the frequency of consumption of certain food groups, such as linking a high consumption of protein to diabetes or linking 'diversity' of different food groups to NCDs

Author Response: The process of DDS is now explained in section 2.3 'Dietary Diversity Score. In this study

6. Why was weight category not included in the co-variates? As obesity is a comorbidity

   Author Response: Weight has a significant impact on the obesity and other NCDs of an individual. As the study has used secondary sources of data and due to a lack of information, we could not include weight data. However, this is included as one of the limitations in the 'Study limitations' section.

7. Why weren't carbohydrates included in the food groups? Since previous studies have linked a high intake of carbohydrates, particularly processed, to a higher incidence of diabetes

   Author Response: The raw data used in this study collects dietary information by using nine specific food groups, as mentioned in the 'Dietary Diversity Score' Section. Therefore, the data does not have any nutrient-specific information, i.e., calorie, protein, fat, carbohydrates, etc., However, the data collects information on carbohydrate-rich food groups such as milk, beans, fruits, etc., which are included in the study.

8. A limitation of the study would be to mention that the 'quantities of protein consumed' were not included rather only frequency 'Our study also shows that the prevalence of diabetes (3.9 percent), heart disease (2.3 percent), and cancer (0.6 percent) is highest among those adults who consumed fish daily than any other category.'

   Author Response: We appreciate the comment and incorporated it. A separate paragraph is added in the 'conclusion' section to include a few limitations of the study, where the third limitation address this particular point.

9. The study is quite general and reads a bit distracting
   Author Response: The manuscript is now edited and revised for more clarity.
